# Evaluating quality of life in frailty: applicability and clinimetric properties of the SarQoL^®^ questionnaire

**DOI:** 10.1002/jcsm.12687

**Published:** 2021-02-28

**Authors:** Anton Geerinck, Médéa Locquet, Olivier Bruyère, Jean‐Yves Reginster, Charlotte Beaudart

**Affiliations:** ^1^ Division of Public Health, Epidemiology and Health Economics University of Liège, World Health Organization Collaborating Centre for Public Health aspects of musculo‐skeletal health and ageing Liège Belgium; ^2^ Biochemistry Department, College of Science King Saud University Riyadh Saudi Arabia

**Keywords:** Frailty, Quality of life, Clinimetrics, Psychometrics, Patient‐reported outcome measure

## Abstract

**Background:**

The SarQoL® questionnaire was specifically designed to measure quality of life (QoL) in sarcopenia. Frailty and sarcopenia have areas of overlap, notably weak muscle strength and slow gait speed, which may mean that the SarQoL could provide a measure of QoL in frailty. This study aimed to evaluate the clinimetric properties of the SarQoL questionnaire in physical frailty using the Fried criteria.

**Methods:**

Analyses were carried out on data from the Sarcopenia and Physical impairment with advancing Age study. Frailty was assessed with the Fried criteria and QoL with the SarQoL, the Short‐Form 36‐Item, and the EuroQoL 5‐Dimension (EQ‐5D) questionnaires. We evaluated discriminative power (with the Kruskal–Wallis analysis of variance test), internal consistency (with Cronbach's alpha), construct validity (through hypotheses testing), test–retest reliability (with the intraclass correlation coefficient), measurement error (calculating standard error of measurement and smallest detectable change), and responsiveness (through hypotheses testing and standardized response mean).

**Results:**

In total, 382 participants were included for the validation and 117 for the responsiveness evaluation. They had a median age of 73 (69–79) years, took 5 (3–8) drugs, and had 4 (3–5) co‐morbidities. There were more women (*n* = 223; 58.4%) than men and, in total, 172 (45%) robust, 167 (44%) pre‐frail, and 43 (11%) frail participants. Discriminative power was confirmed when significantly lower (*P* < 0.001) overall SarQoL scores, and thus also worse QoL, were observed between robust [77.1 (64.35–85.90)], pre‐frail [62.54 (53.33–69.57)], and frail [49.99 (40.45–56.06)] participants. Six of the SarQoL domains performed likewise, with significantly lower scores according to frailty status with Domain 7 (fears) being the exception. Internal consistency was good (*α* = 0.866). Convergent (using Short‐Form 36‐Item and EQ‐5D) and divergent construct validity (using EQ‐5D) was confirmed. Test–retest reliability was excellent [intraclass correlation coefficient = 0.918 (0.834–0.961)], with a standard error of measurement of 3.88 and a smallest detectable change of 10.76 points. We found moderate responsiveness when five of the nine hypotheses were confirmed, coupled with a large effect size for the overall SarQoL score (corrected standardized response mean of −1.44).

**Conclusions:**

The SarQoL questionnaire has adequate clinimetric properties for use with frail patients in clinical practice and trials and could provide data that are more appropriate and detailed than the generic questionnaires currently used.

## Introduction

The World Health Organization declared the period from 2020 to 2030 to be the decade of healthy ageing, which they define as ‘the process of developing and maintaining the functional ability that enables wellbeing in older age’.^1^ This concept is closely linked to the syndrome of frailty, a clinically recognizable state of increased vulnerability in older people, caused by age‐related losses in physiological reserves and function across multiple organ systems, such that the ability to cope with everyday or acute stressors is compromised.^2^

This state of increased vulnerability is associated with negative health outcomes, as evidenced by a recent meta‐analysis, which found an increased likelihood of premature mortality, hospitalization, and institutionalization.^3^ Frailty was also associated with an increased risk for developing disability in both basic and instrumental activities of daily living, an increased risk for physical limitations, dependency, falling, fractures, cognitive decline, decline in lean body mass, and lower life satisfaction.^3^ These outcomes, in combination with an estimated prevalence of 10.7–18%, mean that frailty represents an important burden on public health.^4^, ^5^, ^6^

While hard outcomes such as mortality and hospitalizations remain the primary indicators in research settings, outcomes measuring the subjective experience of patients are becoming as essential part of the arsenal. Health‐related quality of life is one of the main patient‐reported outcome measures used in research, and several studies have already focused on quality of life (QoL) in frailty in the last decade. A 2019 systematic review listed 22 studies that assessed QoL in frailty and which demonstrated that frail participants had worse QoL than robust participants. However, these differences between frail and robust people were only clear for the sub‐concepts of physical functioning and satisfaction with life. For social and environment scales, results were inconsistent between the different questionnaires used, limiting their usefulness in assessing the psychosocial well‐being pre‐frail and frail individuals. In this systematic review, the Short‐Form 36‐Item (SF‐36) was the most frequently used instrument out of the 14 instruments included, followed by the WHOQOL‐BREF, the CASP‐19, and the EUROHIS‐QOL.^7^ Several observations can be made from the results of this systematic review. First, the SF‐36, which was the most frequently used instrument to measure QoL in frailty, is a generic instrument and not adapted to specific populations or diseases.^8^ While generic instruments allow QoL to be compared between a range of populations, specific instruments often possess better construct validity and are more sensitive to changes in QoL over time.^9^ Secondly, the concept of QoL and the components needed to provide a holistic assessment were interpreted differently between each of the QoL questionnaires. While some concepts from the generic QoL questionnaires mentioned previously are shared with the sarcopenia quality of life (SarQoL^®^) questionnaire (i.e. physical and mental health and activities of daily living), others such as ‘body composition’, ‘leisure activities’, and ‘fears’ are unique.

The systematic review did not include frailty‐specific QoL instruments. A QoL instrument specific to the frailty syndrome might improve sensitivity to change in disease‐specific QoL over time in this group.^10^

One such specific questionnaire is the SarQoL questionnaire, developed in 2015 with the aim of measuring health‐related QoL in sarcopenic persons.^11^ The questionnaire was constructed using input from experts, literature review, and crucially, interviews with older, sarcopenic individuals. It has been validated for use with sarcopenic, older, community‐dwelling participants in multiple languages and has consistently been shown to be a valid and reliable instrument, as well as responsive to changes in QoL.^12^, ^13^, ^14^, ^15^, ^16^, ^17^, ^18^, ^19^, ^20^, ^21^

Multiple authors have argued that the conceptual frameworks of frailty and age‐related sarcopenia overlap substantially, notably on the similar clinical manifestations used to diagnose the two conditions. The slowness indicator in the Fried criteria for frailty and the low gait speed indicator used to characterize muscle function in sarcopenia are one area of overlap between the two conditions. Partial overlap exists between weight loss in frailty and muscle loss in sarcopenia, and fatigue/exhaustion in frailty and grip strength in sarcopenia. Some have argued that sarcopenia is equivalent to the physical component of frailty, separate from the cognitive, psychological, sociological, and spiritual components of frailty.^22^, ^23^, ^24^

Because of the overlap between sarcopenia and physical frailty, we considered it worthwhile to explore whether the SarQoL questionnaire could be used in the assessment of QoL in frail and pre‐frail individuals, as diagnosed with the Fried criteria. This study aims to examine the clinimetric properties of the SarQoL questionnaire in robust, pre‐frail, and frail participants of the Sarcopenia and Physical impairment with advancing Age (SarcoPhAge) study.

## Methods

### Population

The analyses described in this manuscript have been carried out using the data collected during the SarcoPhage study. This cohort study followed a sample of community‐dwelling older people for 5 years and has been described in multiple publications ^25^, ^26^, ^27^, ^28^, ^29^. In brief, the SarcoPhAge study recruited a convenience sample of volunteers aged 65 years or older living in the Liège province of Belgium. Participants were recruited from different departments of an outpatient clinic in Liège, as well as through advertisement in the local press. Candidates were not eligible for inclusion in the cohort if they presented with a body mass index (BMI) >50 kg/m^2^ or if they had one or more amputated limbs. No other exclusion criteria were applied. Participants were invited to the research centre once yearly, where they performed physical tests and completed questionnaires.^25^ For the analyses presented here, we used data from Year 1 of follow‐up, except for the evaluation of the responsiveness of the questionnaire, where we used data from the visits carried out at 1 and 5 years into the study.

### Frailty evaluation

In the SarcoPhAge sample, physical frailty was evaluated with the criteria described by Fried *et al*.^30^ The Fried diagnostic criteria evaluate five items to determine whether a person is considered to be robust, pre‐frail, or frail. In this study, the five criteria were measured with the following instruments: weakness was present if handgrip strength measured with hydraulic dynamometer was below the cut‐offs based on gender and BMI, low gait speed was detected by evaluating usual walking speed on a 4 m track (results corrected to 4.5 m track) with cut‐offs based on gender and height, low physical activity was measured with the Minnesota Leisure Time Activity Questionnaire^31^ using gender‐specific cut‐offs for kilocalories used in physical activity in the preceding week, exhaustion was established using two items from the Center for Epidemiological Studies Depression scale,^32^ and weight loss was detected through a self‐reported question on unintentional weight loss of more than 4.5 kg in the past year.^25^, ^30^ For each item, participants were given 1 point if below the cut‐off, and 0 if not, and these item scores were summed for a frailty score between 0 and 5. Participants with zero points were considered robust, a score of 1 or 2 points indicated a pre‐frail state, and subjects with a score of 3 or more points were considered to be frail. A detailed description of the criteria, instruments, and cut‐off values is provided in Supporting Information, *Table* S1.

### Quality of life measurement

The SarQoL questionnaire, the focus of this validation study, is a patient‐reported outcome measure specifically designed to evaluate QoL in older, sarcopenic, community‐dwelling people. There are 55 items in the questionnaire, categorized into seven domains of health‐related dysfunction: (i) physical and mental health, (ii) locomotion, (iii) body composition, (iv) functionality, (v) activities of daily living, (vi) leisure activities, and (vii) fears. A score between 0 (worst QoL) and 100 (best QoL) is provided for each domain, and an overall QoL score (range: 0–100 points) is calculated on the entirety of the questionnaire.^11^ The scoring algorithm is not publicly available, but tools to calculate the scores are available upon request via info@sarqol.org or via the website www.sarqol.org and free for non‐sponsored research. The questionnaire is self‐reported and takes about 15 min to complete. The SarQoL questionnaire has been validated in multiple languages and has been shown to be a valid and reliable instrument.^13^, ^15^, ^16^, ^17^, ^18^, ^19^, ^33^ The questionnaire was shown to be responsive to changes in QoL in a sample of 42 sarcopenic subjects followed over 3 years, and its standard error of measurement (SEM) and smallest detectable change have been calculated in different European populations as well as pooled.^20^, ^21^

Complementary to the SarQoL questionnaire, two generic QoL questionnaires were also completed by each participant to allow the evaluation of the construct validity of the SarQoL questionnaire. The first of these, the SF‐36 questionnaire, measures functional health and well‐being from the patient's perspective, providing eight domain scores (physical functioning, social functioning, role functioning physical, role functioning emotional, vitality, bodily pain, mental health, and general health) and two summary scores (physical and mental), all scored from 0 (worst QoL) to 100 (best QoL) points.^34^ Secondly, the EuroQoL 5‐Dimension 3‐Level (EQ‐5D‐3L) and the associated visual analogue scale (EQ‐VAS) were administered. The EQ‐5D is a generic measure of health status, which records self‐reported problems (none, some, and extreme) on five dimensions (mobility, self‐care, usual activities, pain/discomfort, and anxiety/depression).^35^ Results are reported as an index score (between 0 and 1, with 0 indicating death and 1 indicating perfect health) and a self‐rated health evaluation (between 0, worst imaginable health, and 100, best imaginable health).^36^

### Clinimetric properties

The measurement properties to be included in this validation were selected based on the COSMIN taxonomy and its related documentation.^37^, ^38^ These include known‐groups validity (also known as discriminative power), internal consistency, construct validity (through hypotheses testing), reliability (test–retest), measurement error, and responsiveness. We also looked at the presence of floor and/or ceiling effects and provided the smallest detectable change to aid in the interpretation of the evolution of the SarQoL scores over time.

- Known‐groups validity is based on the hypothesis that two or more groups with distinctive characteristics should logically differ in the construct that is measured.^39^ In the context of this study, the hypothesis is that robust participants should have higher QoL scores than pre‐frail and frail participants, which would mean that the SarQoL questionnaire can discriminate between the three frailty profiles.
- Internal consistency quantifies the degree of interrelatedness between the items in the questionnaire, that is, whether all items in the SarQoL measure the same underlying construct (QoL).^37^, ^38^
- Construct validity is used to assess whether the questionnaire under investigation actually measures what it theoretically aims to measure. This is performed by comparing the questionnaire with other questionnaires (or subscales of) that should, in theory, measure the same construct (convergent validity) or a different construct (divergent validity).^37^, ^38^ In this study, we utilized the same eight hypotheses on the strength of association between the overall QoL score of the SarQoL questionnaire and domains of the SF‐36 and EQ‐5D that were used in previous validations.^13^, ^14^, ^15^, ^16^, ^17^, ^18^, ^19^, ^33^
- The test–retest reliability of the questionnaire shows whether the scores measured by the SarQoL questionnaire remain stable between multiple administrations, on the condition that the participants' health state also remains stable.^37^, ^38^ To measure this, the SarQoL questionnaire was administered twice, with an approximate interval of 2 weeks in between, and participants provided information on the stability of their health. Because of the different objectives of the study that collected the data analysed in this article, only the 43 participants who were diagnosed as sarcopenic with the European Working Group on Sarcopenia in Older People criteria were invited, at the time of the original validation study, to participate in the retest part, with 30 providing usable data.^13^ ReliabilityJCSM_12687 is also demonstrated by the SEM, which provides a measure of the dispersion of observed scores around the ‘true’ score from repeated measurements. The smallest detectable change provides the value for the minimum change in QoL scores that needs to be observed to be certain that the measured change in QoL is real and not possibly due to measurement error.^38^
- Floor and ceiling effects indicate that the range of the scale is too narrow and that extreme profiles cannot be accurately measured. They are present when >15% of the participants obtain either the highest or lowest score.
- The last clinimetric property investigated was the responsiveness of the questionnaire, that is, its capacity to detect change over time, between the first and fifth years of the SarcoPhAge study.^21^ We used the same methodology as in a previous evaluation of the responsiveness of the SarQoL, which was combination of hypothesis testing and effect size evaluation.^21^ In short, we evaluated nine hypotheses (see *Table* 5) on the theorized strength of correlation between the changes observed with the SarQoL questionnaire between Year 1 and Year 5 and the changes observed with (domains of) the SF‐36, EQ‐5D, and EQ‐VAS. The results were interpreted with the criteria from de Boer *et al*., which indicate that a questionnaire has high responsiveness if at least 75% of hypotheses are confirmed, moderate when at least 50–75% are confirmed, and poor responsiveness when less than 50% are confirmed.^40^ In this analysis, we included all participants for whom we had valid data at Year 1 and Year 5. For the second method, we calculated standardized response means (SRMs) (a measure of effect size), which reflect the magnitude of change measured by the SarQoL and by the other questionnaires used in this study. Larger effect sizes indicate that the questionnaire possesses better responsiveness.^41^ Because this method is based on the assumption that a change in health status has occurred, we could only include those participants for whom we had valid data at Year 1 and Year 5 and whose frailty status changed in the years between evaluations. The change in frailty status is used here as a proxy measure of change in health status, and we hypothesize that a change in frailty status will be reflected in the observed change in QoL.

### Statistical analysis

Normality of distribution for quantitative variables was tested with the Shapiro–Wilk test, by comparing mean and median and by evaluating the histogram and Q–Q plot. Continuous variables following a Gaussian distribution are reported as mean ± standard deviation, while those who do not are reported as median (25th–75th percentile). Nominal variables are reported as absolute (*n*) and relative (%) frequencies. The evaluation of differences between groups for nominal variables was carried out using Pearson's *χ* ^2^ test. All results were considered significant at 5% level (*P* ≤ 0.05), except for pairwise comparisons between the robust, pre‐frail, and frail groups, which were considered significant at *P* ≤ 0.017 (*P*‐value adjusted for the number of comparisons: *α* = 0.05/3). IBM SPSS Statistics Version 25 for Windows (IBM Corp., Armonk, NY) was used for all statistical manipulations.

- Analysis of continuous variables to determine the known‐groups validity between the three frailty categories was carried out with the analysis of variance test if distributions in all groups were Gaussian and with the Kruskal–Wallis test if they were not. Paired comparisons were carried out with multinomial regression analysis, so as to obtain *P*‐values for the differences between the robust, pre‐frail, and frail groups.
- Internal consistency was determined with Cronbach's alpha test, where a value between 0.70 and 0.95 indicates good internal consistency.^42^
- Associations between two continuous variables (such as used in the hypotheses for evaluating construct validity and responsiveness) were examined with Pearson's or Spearman's correlations, depending on normality of distribution.
- The test–retest reliability was quantified by calculating the intraclass correlation coefficients (ICCs) (two‐way mixed model − absolute agreement type) between the scores from the first and the second administrations. ICCs greater than 0.7 indicate acceptable reliability.^38^ The SEM was calculated by dividing the standard deviation of the difference between the scores from the first administration and those of the second administration by the square root of 2. This gives the following formula: SEM = (SD_(test score − retest score)_∕√2). The smallest detectable change is derived from the SEM value, by the following formula: 1.96 * √2 * SEM.
- Floor and ceiling effects were evaluated following inspection of the frequency tables.
- Finally, SRMs, a measure of effect size and used to evaluate responsiveness, were calculated by dividing the mean difference between the SarQoL scores from the first year and the fifth year of the SarcoPhAge study by the standard deviation of the differences between these paired values. The SRM values were subsequently transformed with the formula SRM/ √2∕√(1 − *r*), where ‘*r*’ signifies the correlation between Year 1 and Year 5 scores.^43^ The corrected SRM values can now be interpreted with the thresholds formulated by Cohen *et al*., where SRM < 0.20 is trivial effect, 0.20 ≤ SRM < 0.50 is a small effect, 0.50 ≤ SRM < 0.80 is a moderate effect, and SRM ≥ 0.80 is considered a large effect.^44^

## Results

### Clinical characteristics

In total, 382 subjects were eligible for inclusion at the first follow‐up visit of the SarcoPhAge study. These subjects had a median age of 73 (69–78) years old, were slightly overweight at a median BMI of 27 (24–30) kg/m^2^, took a median of 5 (3–8) drugs, and had a median of 4 (3–5) co‐morbidities. There were slightly more women (*n* = 223; 58.4%) than men in the sample. The median grip strength was 39 (33–45) kg for men and 21 (17.5–25) kg for women. Lastly, the median gait speed in the complete sample was 1.09 (0.91–1.28) m/s.

All 382 participants were evaluated for frailty with the Fried criteria, and we found 172 (45%) robust, 167 (44%) pre‐frail, and 43 (11%) frail individuals. Clinical characteristics were significantly different between these three groups. Frail participants were older than pre‐frail participants, who, in turn, were older than robust individuals (*P* < 0.001). The same dynamic was present for BMI (with frail participants having the highest BMI; *P* < 0.001), drug consumption (with frail participants taking the most drugs; *P* < 0.001), and co‐morbidities (with frail participants having the highest number of co‐morbidities; *P* < 0.001). As expected, frail participants had lower grip strength than pre‐frail participants, who, in turn, had lower grip strength than robust people (*P* < 0.001 for men and women). The same was observed for gait speed (with frail participants having the lowest gait speed; *P* < 0.001). Detailed results and pairwise comparisons are available in *Table* 1.

**Table 1:** Clinical characteristics

	All (*n* = 382)	Robust (*n* = 172)	Pre‐frail (*n* = 167)	Frail (*n* = 43)	*P*	*P*	*P*	*P*
					R‐PF^a^	R‐F^b^	PF‐F^c^	Overall^d^
Age (years)	73.18 (68.77–78.47)	70.76 (68.15–76.05)	73.79 (69.45–79.59)	76.52 (71.87–81.82)	<0.001	<0.001	0.031	<0.001
BMI (kg/m^2^)	26.86 (23.74–30.12)	26.24 (23.4–28.68)	27 (23.82–30.43)	28.74 (24.67–34)	0.077	0.001	0.037	0.004
Drug consumption (*n*)	5 (3–8)	5 (3–6)	6 (4–8)	7 (5–10)	<0.001	<0.001	0.079	<0.001
Co‐morbidities *(n)*	4 (3–5)	3 (2–5)	4 (3–6)	4 (3–7)	<0.001	<0.001	0.249	<0.001
Female, *n* (%)	223 (58.4%)	97 (56.4%)	98 (58.7%)	28 (65.1%)				0.580
Grip strength (kg)								
Men	39 (33–45)	40 (37–45)	38 (31–45)	26 (18–32)	0.012	<0.001	<0.001	<0.001
Women	21 (17.5–25)	24 (21–27.5)	19 (16–22)	13 (11.63–17.75)	<0.001	<0.001	<0.001	<0.001
Gait speed (m/s)	1.09 (0.91–1.28)	1.2 (1.05–1.35)	1.06 (0.86–1.23)	0.72 (0.53–0.87)	<0.001	<0.001	<0.001	<0.001

BMI, body mass index; QoL, quality of life.

All results reported as median (25th–75th percentile).

^a^ *P*‐values for pairwise comparison between robust and pre‐frail groups (significant at *P* ≤ 0.017).

^b^ *P*‐values for pairwise comparison between robust and frail groups (significant at *P* ≤ 0.017).

^c^ *P*‐values for pairwise comparison between pre‐frail and frail groups (significant at *P* ≤ 0.017).

^d^ *P*‐values obtained through Kruskal–Wallis analysis of variance test with pairwise comparisons, except for gender (*χ* ^2^ test).

### Known‐groups validity

The overall QoL score measured by the SarQoL questionnaire was significantly different (*P* < 0.001) between the three categories of frailty, following a downward trend with robust participants having the best QoL [77.10 (64.35–85.90)], followed by the pre‐frail participants [62.54 (53.33–69.57)] and with the frail participants presenting with the worst QoL [49.99 (40.45–56.06)]. The differences between the overall QoL scores in these three groups were revealed to be significant in the paired comparisons (all *P* < 0.001).

The QoL scores for the seven domains of health‐related quality of life in the SarQoL questionnaire were also shown to be highly significantly different (*P* < 0.001). An examination of the paired differences showed that only Domain 7 (fears) was not significantly different in the comparison between pre‐frail and frail groups (*P* = 0.119).

The complete results of the known‐groups validity are presented in *Table* 2.

**Table 2:** Discriminative power of the SarQoL^®^ questionnaire in frailty

	Robust (*n* = 172)	Pre‐frail (*n* = 167)	Frail (*n* = 43)	*P*	*P*	*P*	*P*
				R‐PF^a^	R‐F^b^	PF‐F^c^	Overall^d^
Domain 1: physical and mental health	72.2 (59.5–86.63)	59.97 (52.2–68.87)	45.53 (38.87–55.53)	<0.001	<0.001	<0.001	<0.001
Domain 2: locomotion	77.78 (58.33–91.67)	55.56 (47.22–69.44)	41.67 (30.56–52.78)	<0.001	<0.001	0.001	<0.001
Domain 3: body composition	70.83 (55.21–80)	58.33 (50–66.67)	50 (41.67–58.33)	<0.001	<0.001	0.014	<0.001
Domain 4: functionality	82.69 (73.11–91.07)	69.23 (59.62–78.85)	55.36 (48.08–62.50)	<0.001	<0.001	<0.001	<0.001
Domain 5: activities of daily living	76.67 (64.47–85.32)	60.71 (48.33–72.50)	50 (33.33–58.33)	<0.001	<0.001	<0.001	<0.001
Domain 6: leisure activities	66.50 (49.88–66.50)	33.25 (33.25–49.88)	33.25 (16.62–33.25)	<0.001	<0.001	0.011	<0.001
Domain 7: fears	100.00 (87.50–100)	87.50 (87.50–100.00)	87.5 (75.00–87.50)	0.032	0.004	0.119	<0.001
Overall QoL score	77.10 (64.35–85.90)	62.54 (53.33–69.57)	49.99 (40.45–56.06)	<0.001	<0.001	<0.001	<0.001

BMI, body mass index; QoL, quality of life.

All results reported as median (25th–75th percentile).

^a^ *P*‐values for comparison between robust and pre‐frail groups. *P*‐values obtained through multinomial regression analysis adjusted on age, BMI, no. of drugs, no. of co‐morbidities, and gender.

^b^ *P*‐values for comparison between robust and frail groups. *P*‐values obtained through multinomial regression analysis adjusted on age, BMI, no. of drugs, no. of co‐morbidities, and gender.

^c^ *P*‐values for comparison between pre‐frail and frail groups. *P*‐values obtained through multinomial regression analysis adjusted on age, BMI, no. of drugs, no. of co‐morbidities, and gender.

^d^ *P*‐values obtained through Kruskal–Wallis analysis of variance test.

### Internal consistency

The homogeneity of the questionnaire was found to be excellent, with Cronbach's alpha of 0.866, at the upper end of the 0.70 to 0.95 range considered good. This shows that the questionnaire is consistent without showing the increased likelihood for redundancy associated with alpha values greater than 0.95. The influence of individual domains was tested by deleting a single domain at a time. The resulting alpha values ranged from 0.854 to 0.894, indicating that no domain unduly influences the internal consistency.

### Construct validity

Two sets of hypotheses were examined: for the convergent construct validity, we theorized that the overall QoL score of the SarQoL questionnaire measures a construct related to the SF‐36 physical functioning, role limitation due to physical problems, and vitality domains as well as to the EQ‐5D utility score. We therefore expect to find moderate to strong correlations between the SarQoL and these domains. For the divergent construct validity, we theorized that the overall QoL score of the SarQoL questionnaire measures a different construct than the SF‐36 role limitation due to emotional problems and mental health domains, as well as the self‐care and anxiety/depression items of the EQ‐5D. If this is correct, we expect to find weak or non‐existent correlations between the SarQoL and these domains.

The convergent validity of the SarQoL questionnaire in the entire sample was excellent, as evidenced by the confirmation of the four pre‐specified hypotheses and the strong correlations between the overall QoL score of the SarQoL questionnaire and the four domains theorized to measure similar constructs (correlation coefficients between 0.447 and 0.798). When isolating the three frailty groups, the results were largely similar, with the exception of the correlation between the SarQoL and the SF‐36 role limitation due to physical problems domain in the frail group, which dropped from *r* = 0.628 (*P* < 0.001) to *r* = 0.246 (*P* = 0.199).

The results of the divergent construct validity were less straightforward: both in the complete sample and in the three frailty categories, we found moderate to strong correlations between the SarQoL questionnaire and the two domains of the SF‐36 theorized to be measuring a different construct. The hypotheses with the EQ‐5D self‐care and anxiety/depression items were confirmed by weak correlations (respectively, *r* = −0.273; *P* < 0.001 and *r* = −0.257; *P* < 0.001).

The full results, shown in *Table* 3, demonstrate that six out of the eight pre‐specified hypotheses were confirmed, fulfilling the criteria of 75%, which indicates acceptable construct validity.

**Table 3:** Construct validity of the SarQoL^®^ questionnaire in frailty

	Robust (*n* = 172)		Pre‐frail (*n* = 167)		Frail (*n* = 43)		All (*n* = 382)	
	*r*	*P*	*r*	*P*	*r*	*P*	*r*	*P*
**Convergent validity**								
SF‐36 physical functioning	0.761	<0.001	0.693	<0.001	0.608	<0.001	0.798	<0.001
SF‐36 role limitation physical	0.408	<0.001	0.611	<0.001	0.246	0.199	0.628	<0.001
SF‐36 vitality	0.595	<0.001	0.499	<0.001	0.695	<0.001	0.678	<0.001
EQ‐5D utility score	0.305	<0.001	0.311	<0.001	0.564	0.001	0.447	<0.001
**Divergent validity**								
SF‐36 role limitation emotional	0.400	<0.001	0.473	<0.001	0.015	0.936	0.503	<0.001
SF‐36 mental health	0.588	<0.001	0.489	<0.001	0.392	0.035	0.554	<0.001
EQ‐5D self‐care	^a^	^a^	−0.207	0.011	−0.278	0.145	−0.273	<0.001
EQ‐5D anxiety/depression	−0.161	0.070	−0.222	0.010	−0.489	0.007	−0.257	<0.001

^a^ All 172 robust subjects responded identically on the EQ‐5D self‐care question.

### Reliability

One of the 30 participants was not evaluated for physical frailty, which means that test–retest data were available for 29 participants, of which 4 (13.8%) were robust, 18 (62.1%) were pre‐frail, and 7 (24.1%) were frail. An ICC of 0.918 [95% confidence interval (CI) = 0.834–0.961] was found for the overall QoL score of the SarQoL questionnaire, demonstrating excellent test–retest reliability. The ICCs for the individual domains showed acceptable (ICC > 0.7) reliability for all but two domains: Domain 6, leisure activities [ICC = 0.391 (95% CI = 0.029–0.660)], and Domain 7, fears [ICC = 0.318 (95% CI = −0.055 to 0.612)]. Detailed results for the test–retest reliability are reported in *Table* 4.

**Table 4:** Test–retest reliability of the SarQoL^®^ questionnaire in frailty

	ICC	95% CI	SEM	SDC
Domain 1: physical and mental health	0.764	0.558–0.881	7.06	19.57
Domain 2: locomotion	0.850	0.706–0.926	7.92	21.94
Domain 3: body composition	0.700	0.454–0.847	8.81	24.41
Domain 4: functionality	0.879	0.759–0.941	5.50	15.25
Domain 5: activities of daily living	0.812	0.638–0.907	6.78	18.80
Domain 6: leisure activities	0.391	0.029–0.660	13.82	38.30
Domain 7: fears	0.318	−0.055 to 0.612	14.32	39.68
Overall QoL score	0.918	0.834–0.961	3.88	10.76

CI, confidence interval; ICC, intraclass correlation coefficient; QoL, quality of life; SDC, smallest detectable change; SEM, standard error of measurement.

The SEM in this sample was calculated to be 3.88 points, leading to a smallest detectable change of 10.76 points. In practical terms, the overall QoL score of an individual participant would have to change by 10.76 points to be able to be sure that the observed change in QoL is due to a real change in QoL in the patient. SEM and smallest detectable change for the individual domains are reported in *Table* 4.

### Floor and ceiling effects

None of the 382 participants obtained the lowest (0) or the highest (100) score possible for the overall QoL score of the SarQoL^®^ questionnaire, showing the absence of floor and ceiling effects in the summary score.

### Responsiveness

Out of the 382 participants who provided usable data at the first year of the SarcoPhAge study, 235 remained in the study at the fifth year of follow‐up and were included in the responsiveness evaluation. Of these 235, a further 117 changed in terms of their frailty status between the first and fifth years of the study, and these were included in the analysis of responsiveness through the evaluation of effect size (SRMs).

We examined nine hypotheses used in an earlier study of the responsiveness of the SarQoL questionnaire, on the theorized correlation between changes measured by the SarQoL questionnaire and by other questionnaires. We were able to confirm five out of nine hypotheses but had to reject Hypothesis 1 (∆SarQoL overall score and ∆SF‐36 general health), Hypothesis 4 (∆SarQoL overall score and ∆EQ‐VAS), Hypothesis 5 (∆SarQoL Domain 1 and ∆SF‐36 general health), and Hypothesis 6 (∆SarQoL Domain 1 and ∆EQ‐VAS). According to the criteria formulated by De Boer *et al*., this indicated that the SarQoL questionnaire possesses moderate responsiveness because 45% of the hypotheses have been refuted. The details of the hypotheses and the observed correlations can be found in *Table* 5.

**Table 5:** Evaluation of responsiveness with hypotheses

Hypothesis	Expected strength of correlation	Observed correlation		Confirmation/rejection
		*r*	*P*‐value	
1. ∆SarQoL^®^ overall score and ∆SF‐36 general health domain are correlated.	*r* > 0.4	0.389^a^	<0.001	Rejected
2. ∆SarQoL^®^ overall score and ∆SF‐36 vitality domain are correlated.	*r* > 0.3	0.460^b^	<0.001	Confirmed
3. ∆SarQoL^®^ overall score and ∆SF‐36 physical functioning domain are correlated.	*r* > 0.5	0.690^a^	<0.001	Confirmed
4. ∆SarQoL^®^ overall score and ∆EQ‐VAS are correlated.	*r* > 0.4	0.226^a^	<0.027	Rejected
5. ∆SarQoL^®^ Domain 1 (physical and mental health) and ∆SF‐36 general health domain are correlated.	*r* > 0.3	0.139^a^	0.176	Rejected
6. ∆SarQoL^®^ Domain 1 (physical and mental health) and ∆EQ‐VAS are correlated.	*r* > 0.3	0.142^a^	0.166	Rejected
7. ∆SarQoL^®^ Domain 2 (locomotion) and ∆SF‐36 physical functioning domain are correlated.	*r* > 0.4	0.539^a^	<0.001	Confirmed
8. ∆SarQoL^®^ Domain 4 (functionality) and ∆SF‐36 physical functioning domain are correlated.	*r* > 0.5	0.601^a^	<0.001	Confirmed
9. ∆SarQoL^®^ Domain 5 (activities of daily living) and ∆SF‐36 physical functioning domain are correlated.	*r* > 0.5	0.617^a^	<0.001	Confirmed

Δ = change over 4 years.

^a^ Spearman correlation.

^b^ Pearson correlation.

We also evaluated responsiveness with the metric of effect size. We calculated SRMs for all domains and summary scores of the SarQoL, SF‐36, and EQ‐5D questionnaires. The complete results are reported in *Table* 6. We can observe that the SRM of the SarQoL overall score (corrected SRM = −1.14) is much larger than the SF‐36 PCS (corrected SRM = −0.634), the EQ‐5D index (corrected SRM = 0.064), and the EQ‐VAS (corrected SRM = −0.267). Globally, the SarQoL questionnaire had small effect sizes for three domain scores, moderate for 2 and large for 1. The SF‐36 obtained small effect sizes for five domains and the MCS, and moderate effect sizes for three domains and the PCS.

**Table 6:** Standardized response means

Domains	Corrected SRM	Interpretation^a^
1. ∆SarQoL^®^ D1 physical and mental health	−0.383	Small
2. ∆SarQoL^®^ D2 locomotion	−0.755	Moderate
3. ∆SarQoL^®^ D3 body composition	−0.315	Small
4. ∆SarQoL^®^ D4 functionality	−0.940	Large
5. ∆SarQoL^®^ D5 activities of daily living	−0.883	Large
6. ∆SarQoL^®^ D6 leisure activities	−0.255	Small
7. ∆SarQoL^®^ D7 fears	−0.070	Trivial
8. ∆SarQoL^®^ overall score	−1.144	Large
9. ∆SF‐36 physical functioning	−0.749	Moderate
10. ∆SF‐36 social functioning	−0.204	Small
11. ∆SF‐36 role limitations due to physical health	−0.301	Small
12. ∆SF‐36 role limitations due to emotional problems	−0.251	Small
13. ∆SF‐36 mental health	−0.274	Small
14. ∆SF‐36 vitality	−0.577	Moderate
15. ∆SF‐36 bodily pain	−0.490	Small
16. ∆SF‐36 general health	−0.693	Moderate
17. ∆SF‐36 physical component summary	−0.634	Moderate
18. ∆SF‐36 mental component summary	−0.224	Small
19. ∆EQ‐5D utility index	0.064	Trivial
20. ∆EQ‐VAS	−0.267	Small

SRM, standardized response mean.

SRMs are calculated by dividing the mean difference between scores from the first year and the first year by the standard deviation of the differences between these paired values. The SRM values were subsequently corrected with the formula SRM∕√2∕√(1 − *r*), where ‘*r*’ signifies the correlation between Year 1 and Year 5 scores. Δ = change over 4 years.

^a^ Interpretation of corrected SRMs: 0.20 ≤ SRM < 0.49 = small change; 0.50 ≤ SRM < 0.79 = moderate change; and SRM ≥ 0.80 = large change.

## Discussion

This study examined whether the SarQoL questionnaire could be used as a disease‐specific instrument to measure health‐related QoL in frailty. The psychometric results presented in this article indicate that it has adequate measurement properties when used with the Fried frailty criteria. This means that the SarQoL could be a new option for researchers seeking to evaluate QoL in populations characterized by the presence of pre‐frailty and/or frailty.

This study demonstrated that the SarQoL questionnaire can discriminate between robust, pre‐frail, and frail subjects, with declining QoL scores according to the category of frailty, and that it can do so over a wide range of concepts. The systematic review by Crocker *et al*. highlighted that (sub)scales measuring physical aspects of QoL were broadly able to discriminate between robust and frail people but reported inconsistent results for other aspects of QoL.^7^ Therefore, it is encouraging to see that the SarQoL questionnaire is able to discriminate on more than just the physical aspects of QoL and that it brings extra precision in being able to discriminate between robust, frail, and pre‐frail individuals. A note of caution is warranted with regard to Domain 7, where only the comparison between robust and frail participants yielded significantly different QoL scores. This domain should not be interpreted in a vacuum but taking into account the other domain scores and the overall QoL score.

The internal consistency was shown to be high (*α* = 0.866), indicating that the domains in the questionnaire are highly interrelated and measure the same construct, QoL. Mixed results were obtained in the evaluation of the construct validity of the questionnaire. All four hypotheses on the convergent validity were confirmed, but two out of the four hypotheses for divergent validity were rejected. The two rejected hypotheses, where we found stronger correlations than expected, were between the overall QoL score of the SarQoL questionnaire and the mental health and role limitations due to emotional problems domains of the SF‐36. It may be that our hypotheses are erroneous and that these two domains are conceptually closer to the SarQoL questionnaire than we theorized. One correlation of particular interest is between the SarQoL overall QoL score and the SF‐36 role limitation due to physical limitations in the frail group (*r* = 0.246), because it is significantly lower than the correlation coefficients in the robust (*r* = 0.408) and pre‐frail groups (*r* = 0.611). Upon further investigation, this discrepancy is linked to the significant floor effect in this SF‐36 domain, where 16 of the 30 participants obtain the lowest score possible.

The test–retest reliability of the questionnaire was excellent, with an ICC of 0.918 (95% CI = 0.834–0.961) for the overall score. However, because the original study only contacted the participants diagnosed as sarcopenic with the European Working Group on Sarcopenia in Older People criteria to enter the evaluation of the test–retest reliability, there were only data available for 29 participants. So, while this is a result that indicates good test–retest reliability, with an elevated ICC and a relatively small CI, these results should be confirmed in a larger sample and in particular samples with sufficient pre‐frail and frail participants to calculate ICC's for these particular groups. Because the SEM and the smallest detectable change are based on the test–retest data, this same remark also applies to these two indicators. It should also be noted that, in this study, Domain 6 (leisure activities) and Domain 7 (fears) did not demonstrate adequate reliability. We hypothesize that this may because of the low sample size in combination with the low number of items for these two domains (two items for Domain 6 and four items for Domain 7), which causes any difference between the responses between the first and second administration of the questionnaire to be exaggerated in the scores.

We examined the ability of the SarQoL questionnaire to detect a change in QoL. We found moderate responsiveness through the confirmation of five out of nine hypotheses on the correlation between changes in QoL observed by the SarQoL questionnaire and by other questionnaires. It is possible that the rejection of several hypotheses is linked to the lower SRMs found between the different questionnaires. In fact, the SRM of the overall QoL score of the SarQoL questionnaire is markedly stronger at SRM = −1.144 compared with the strongest effect size of the SF‐36, which was the physical functioning subscale at SRM = −0.749. It may be that the rejection of some hypotheses was thus caused not by poor responsiveness of the SarQoL questionnaire but by smaller effect sizes found by the SF‐36. Similarly, for the EQ‐VAS, we found a small effect at SRM = −0.267 and the rejection of two hypotheses associated with this instrument. Here also, this may be more linked to the performance of the EQ‐VAS in combination with the 4 year interval between the assessments. It is highly likely that an instrument such as the EQ‐VAS would be influenced by response shift, which is defined as a change in the self‐evaluation of the meaning of a target construct caused by reconceptualization of the construct, a reprioritization of the participants' values, or a recalibration of the respondents' internal standards of measurement.^38^, ^45^ Overall assessments, such as the EQ‐VAS, which asks the respondent to indicate on a scale from 0 to 100 ‘how good or bad your health state is today’, are more vulnerable to response shift because they require careful consideration and interpretation of the question. The participants had to evaluate for themselves the meaning of the concept ‘health state’ and what is considered ‘good’ and ‘bad’ and assign a numerical value to this, leaving open the possibility of reconceptualization, reprioritization, or recalibration.^46^ Researchers investigating changes in QoL over time or pre‐intervention/post‐intervention should make the overall QoL score of the SarQoL questionnaire their main outcome, given that it has the highest SRM and the smallest detectable change. If a significant change in overall QoL is found, further analyses of the individual domains could be useful in indicating on what domains a participant's QoL has changed.

Because this study used data collected during a previous study, we were unable to investigate and quantify the content validity of the SarQoL questionnaire in a population of frail, older, community‐dwelling individuals. In the development of the questionnaire, content validity had been put at the heart of the process by soliciting, at each step of the item generation and selection process, input from multiple sarcopenic persons.^11^ In this study, we were unable to provide this information from frail individuals. However, some authors have theorized that sarcopenia, the target condition for which the SarQoL questionnaire was developed, constitutes one of the main components of the clinical frailty syndrome, all the while recognizing that frailty should not be limited to physical manifestations but should also incorporate psychological, cognitive, emotional, social, and spiritual factors.^24^, ^47^ Currently, to our knowledge, the only questionnaire that measures QoL and that is specifically designed with and for older frail persons is the Geriatric Quality of Life Questionnaire.^48^ However, the developers left the definition of what constitutes the ‘frail elderly’ up to the appreciation of the clinicians responsible for recruitment, instead of a recognized diagnostic tool. While the SarQoL questionnaire was not specifically developed for frailty, the shared characteristics between sarcopenia and frailty mean that it should be able to provide a precise measurement of the physical weakness aspect of frailty. Apart from the physical domains, the SarQoL has also incorporated items on mental health, body image, sexuality, activities of daily living, leisure activities, and fears, making for a multidimensional framework of QoL.

Healthy ageing is already high on the agenda for most health systems in both Western and Asian countries and will only gain in importance as the number of older people increases.^49^ Concepts such as frailty, sarcopenia, or the construct recently proposed by the World Health Organization called Intrinsic Capacity, which is a composite of all the physical and mental capacities of an individual, may play an important role in any future medical approach.^50^ Whatever approach is adopted, it must take in the perspective and priorities of the target population, and QoL can be an important metric for this. Having valid, reliable, and precise instruments to measure QoL that can pick up on the impact of a specific target condition is a prerequisite to be able to rely on QoL instruments to provide information on the patients' lived experience.

There are some limitations to this study. First off, we adopted the frailty criteria developed by Fried *et al*., but other diagnostic approaches are available, such as the Rockwood Clinical Frailty Scale or the IF‐VIG, among others.^51^, ^52^, ^53^ Although all these approaches purportedly measure the same concept, frailty, we cannot be sure that the results on the validity and reliability of the SarQoL would have been the same if we had applied other diagnostic approaches. Secondly, our sample of robust, pre‐frail, and frail participants is not necessarily representative of frailty in the wider community. Because these data were collected within a study that recruited volunteers, and which asked those volunteers to make several trips to the research centre, it is likely that the SarcoPhAge study recruited a sample that was in better overall condition, and that had better mobility, than a representative sample of pre‐frail and frail participants. While this study has shown that the SarQoL questionnaire is a valid and reliable tool in frailty, additional investigations in samples with a different make‐up need to confirm these results. Lastly, while the overall sample size was more than adequate for a psychometric study, the test–retest sample is relatively small with only 29 participants. This steep reduction from the overall sample size is a result of the fact that only a subset of participants was invited to complete the questionnaire a second time. However, because we have the 95% CI around the ICC, we can judge that most values have adequate precision, apart from Domains 6 and 7.

In conclusion, the study evaluated the validity and reliability of the SarQoL questionnaire in frailty and found that it is a valid and reliable tool for the assessment of QoL. Because of the shared mechanism of physical weakness between sarcopenia and frailty, the SarQoL questionnaire can provide more specific information on QoL in frailty than the generic questionnaires available.

### Ethical standards

This study performed a secondary analysis of previously collected data. Therefore, no specific approval of a medical ethics committee was sought. The SarcoPhAge study was approved by the Medical Ethics Committee of the University Hospital of Liège, and all participants provided written informed consent, in compliance with the 1964 Declaration of Helsinki and its subsequent amendments.

## Author contributions

All authors participated in the conception, design, and redaction of the manuscript, as well as read and approved the final version of the manuscript. The authors of this manuscript certify that they comply with the ethical guidelines for authorship and publishing in the *Journal of Cachexia, Sarcopenia and Muscle*.^54^

## Funding

A.G. is supported by a FRIA grant from the Fonds De La Recherche Scientifique ‐ FNRS.

## Conflict of interest

C.B., O.B., and J.‐Y.R. are shareholders of SarQoL sprl. A.G. and M.L. declare that they have no conflicts of interest.

## Supporting information


**Table S1.** Frailty criteria and diagnosis
