# Supplementary material for: Evaluating quality of life in frailty: applicability and clinimetric properties of the SarQoL® questionnaire
Source: J Cachexia Sarcopenia Muscle. 2021 Feb 28;12(2):319–30. doi: 10.1002/jcsm.12687 (PMC8061363; doi:10.1002/jcsm.12687)
Supplement: Supplementary file 1 — Table S1. Frailty criteria and diagnosis [file JCSM-12-319-s001.docx]

| Supplemental table: Frailty criteria and diagnosis | | |
| --- | --- | --- |
| Criteria | Cut-off | Scoring |
| Involuntary weight-loss | >4.5 kg in 1 year (if during follow-up visit: more than 5% weight loss since previous visit) | Yes=1  No=0 |
| Handgrip strength | Dominant hand, 3 repetitions, highest value  Men:   - BMI ≤24 => GS ≤29 kg - BMI 24.1 - 26 => GS ≤30 kg - BMI 26.1 - 28 => GS ≤30 kg - BMI >28 => GS ≤32 kg   Women:   - BMI ≤23 => GS ≤17 kg - BMI 23.1 - 26 => GS ≤17.3 kg - BMI 26.1 - 29 => GS ≤18 kg - BMI >29 => GS ≤21 kg | Yes=1  No=0 |
| Exhaustion  “How many times during the past week have you felt like:   1. Everything I do requires effort. 2. I ‘m not going to be able to continue like this.” | A & B  0= rare or never (<1 day)  1= sometimes (1-2 days)  2= occasionally (3-4 days)  3= often (5-7 days) | If the participant gives response 2 or 3 for one or both questions, score 1. |
| Gait speed over 4.5m | Time to walk 4.5m recalculated from SPPB results on a 4m track.  Men:   - Height ≤173 cm => ≥7 sec - Height >173 cm => ≥6 sec   Women   - Height ≤159 cm => ≥7 sec - Height >159 cm => ≥6 sec | Yes=1  No=0 |
| Physical activity level (Minnesota Leisure Time Activity questionnaire) | Men: <283 KCAL/week  Women: <270 KCAL/week | Yes=1  No=0 |
